# Sexual dysfunction after elective spine surgery: A systematic review identifying knowledge gaps and clinical implications

**DOI:** 10.1016/j.bas.2026.106130

**Published:** 2026-06-17

**Authors:** Sara Merkaj, Anton Früh, Claudius Jelgersma, Peter Vajkoczy, Lars Wessels, Vanessa Hubertus

**Affiliations:** a Department of Neurosurgery, Charité – Universitätsmedizin Berlin, Corporate Member of Freie Universität Berlin, Humboldt-Universität zu Berlin, and Berlin Institute of Health, Campus Charité Mitte, Luisenstraße 64, Berlin, 10117, Germany; b Berlin Institute of Health (BIH) - Charité Clinician Scientist Program, Berlin Institute of Health, Anna-Louisa-Karsch-Straße 2, Berlin, 10178, Germany

**Keywords:** Sexual dysfunction, Spine surgery, Elective, Systematic review

## Abstract

**Introduction:**

Spine surgery is one of the most frequently performed surgical procedures globally, with increasing numbers particularly among older patients. Sexual function significantly impacts quality of life, yet sexual dysfunction following spine surgery remains underexplored.

**Research question:**

This systematic review aims to summarize current research on sexual dysfunction following spine surgery, identify knowledge gaps, and clinical implications.

**Methods:**

A systematic literature search was conducted following the PRISMA-DTA guidelines. Relevant articles were identified utilizing MEDLINE and PubMed database focusing on studies reporting sexual function outcomes after elective spine surgery. Fifteen studies meeting the inclusion criteria were analyzed for study type, sample size, surgical approach, and sexual function outcomes.

**Results:**

The majority of studies were retrospective and single-center with small sample sizes. About 62% reported no decline or improvement in sexual function after surgery, while 38% documented a decrease. Surgical approaches and patient populations varied widely. Most studies utilized the Oswestry Disability Index (ODI) to address sexual activity, though its limitations in capturing nuanced sexual dysfunction were noted. Research addressing sexual dysfunction on a deeper level, including psychological and gender-specific sexual health remained scarce.

**Discussion and conclusion:**

Evidence on sexual dysfunction following spine surgery currently is heterogeneous and limited. There is a critical need for high-quality, prospective, gender-specific research employing comprehensive and validated sexual function assessments. The routine evaluation of sexual function holds the potential to enhance understanding, improve patient counseling, postoperative care, and overall quality of life.

## Highlights

• Sexual dysfunction after elective spine surgery is an under-investigated but clinically significant issue impacting patient quality of life.
• Existing studies are limited by methodological heterogeneity, low evidence quality, and reliance on non-specific measurement systems of sexual dysfunction.
• Gender-specific factors and the complex dimensions of sexual health are inadequately addressed in current literature.
• Future research should focus on multicenter, longitudinal studies using validated sexual function–specific tools to the identification of at-risk patients and guide the development of tailored interventions.

## Abbreviations

ASD: Adjacent Segment Disease
ALIF: Anterior Lumbar Interbody Fusion
CSFQ-14: Changes in Sexual Function Questionnaire-14
IIEF-5: EQ-5D and the International Index of erectile function
EQ-5D: EuroQol-5D
FSDS: Female Sexual Distress Scale
FSFI: Female Sexual Function Index
HRQoL: Health-related quality of life
LLPF: Long-segment posterior fixation
LSS: Lumbar Spinal Stenosis
ODI: Oswestry Disability Index
QOL: Quality of Life
SSPF: Short-segment posterior fixation
SF: Sexual Function
SF-12: 12-Item Short-Form Health Survey
TDR: Total Disc Replacement

## 1 Introduction

Spine surgery counts as some of the most commonly performed surgical procedures worldwide. According to inpatient data from the AOK health insurer in Germany, the number of spinal procedures had an 82% rise over the last 10 years. The most significant increase was observed in "bone decompression" surgeries, which rose by 280% (Petzold et al., 2018). These trends also reflect on current statistics, for example, the incidence of posterior spinal fusion is projected to increase by ∼83% by 2060 (Heck et al., 2023). Consequently, the burden of associated side effects and functional impairments is also rising.

Sexual function plays a significant role in a person's quality of life (QOL) (Albright et al., 2015; Kiani et al., 2024; Lim-Watson et al., 2022). Following spine surgery, several factors may influence sexual function, including neurological, psychological, and mechanical components (Albright et al., 2015; Krassioukov and Elliott, 2017; Seftel, 2017). Various patient-reported outcome measures have been developed to assess condition-specific disabilities, such as the Oswestry Disability Index (ODI) (Fairbank, 2014) or EuroQol-5D (EQ-5D) (EuroQol, 1990). The ODI consists of ten items evaluating impairments related to lumbar spine conditions, with item eight specifically addressing the condition's impact on sexual activity. Responses range from "My sex life is normal and causes no extra pain" to "Pain prevents any sex life at all," providing insight into the degree of disability associated with lumbar spine disorders (Fairbank and Pynsent, 2000). However, current outcome measures predominantly emphasize pain-related dysfunction. To our knowledge, there are no established tools focusing specifically on assessing sexual dysfunction following spine surgery.

A growing body of research has emerged on this topic in recent years, examining various aspects of sexual function and dysfunction following spine surgery. Studies have explored sexual function after anterior lumbar surgery in females (Wuertz-Kozak et al., 2019), risk factors for worsening sexual function postoperatively (Nakajima et al., 2021), and the impact of spine surgery on sexual activity in general (Malik et al., 2018). Nevertheless clinical investigations thereby primarily focused on physiological factors contributing to sexual dysfunction, such as erectile dysfunction (Gempt et al., 2010) and delayed ejaculation (Bhambhvani et al., 2022). Additionally, research has been conducted on female sexual dysfunction in patients with various spine pathologies following surgical intervention (Moscicki and Bachmann, 2022). Overall, studies in this field remain heterogeneous, utilizing diverse methodologies and perspectives. Despite this emerging evidence, there is still minimal emphasis on sexual function in routine clinical assessment and postoperative care.

In recent years, there has been an increasing emphasis on patient-based outcomes (Legato et al., 2016). However, a significant knowledge gap remains in our understanding of sexual function before and after spine surgery and factors affecting these changes (Nakajima et al., 2021). Moreover, current measurement systems are insufficient to assess the various aspects of sexual health, and there is an urgent need for further exploration to guide future research and clinical practice in this area.

This study aims to systematically review and summarize current research on sexual dysfunction following elective spine surgery, analyze emerging trends and key research questions, and provide an overview of areas for improvement and future directions. By raising awareness on this topic within the medical community, this study seeks to contribute to enhancing patient care and improving quality of life following spine surgery.

## 2 Methods

This study followed the guidelines outlined in the PRISMA (Preferred Reporting Items for Systematic reviews and Meta-Analyses) statement (Page et al., 2021). A systematic literature search was conducted in MEDLINE and PubMed on March 1, 2025. The search used keywords and controlled vocabulary, including [“sexual function” AND “spine surgery”]. The initial search yielded 530 English-language articles spanning all publication years. Only peer-reviewed studies were imported for text screening and further assessed for relevance. Duplicate studies were identified and removed.

**Fig. 1:**
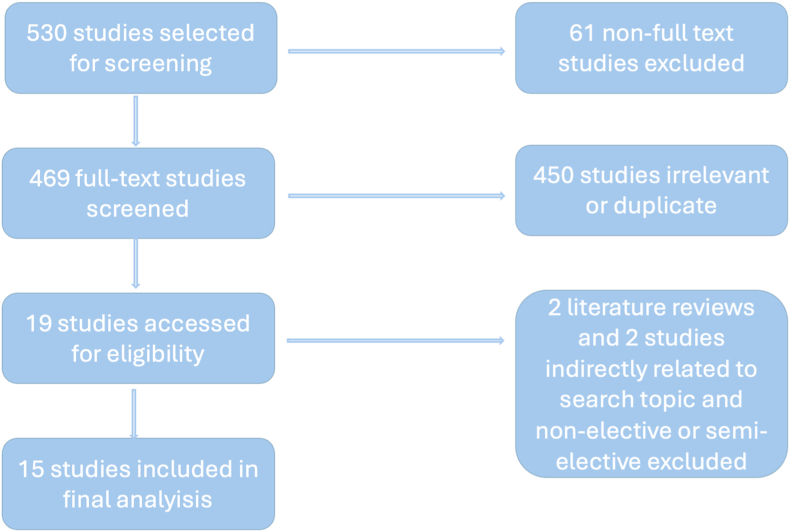
PRISMA Flow Chart of the selection process.

The aim of this study was to explore the examination and occurrence of sexual dysfunction in patients undergoing elective spine surgery without complaints regarding sexual function prior to surgery. To ensure a focused analysis, the following exclusion criteria were established: (1) abstract-only publications; (2) non-primary literature; (3) non-English studies; (4) studies unrelated to the impairment of sexual function after spinal surgery, (5) studies on non-elective or semi-elective spine surgeries. A PRISMA flow diagram illustrating the study selection process is shown in Fig. 1.

Data was extracted using a standardized Microsoft Excel (Microsoft Corporation) form. The following data points were extracted: title and first author, year of publication, study type, number of participants, main research question, key findings. All identified articles were screened for relevance and adherence to inclusion criteria by three experienced physicians. Upon initial abstract screening, full-text articles were assessed, selected and confirmed. Discrepancies were resolved by majority decisions. Data was analyzed using descriptive statistics with R-Studio (Version, 2024.12), and Microsoft Excel.

## 3 Results

**Fig. 2:**
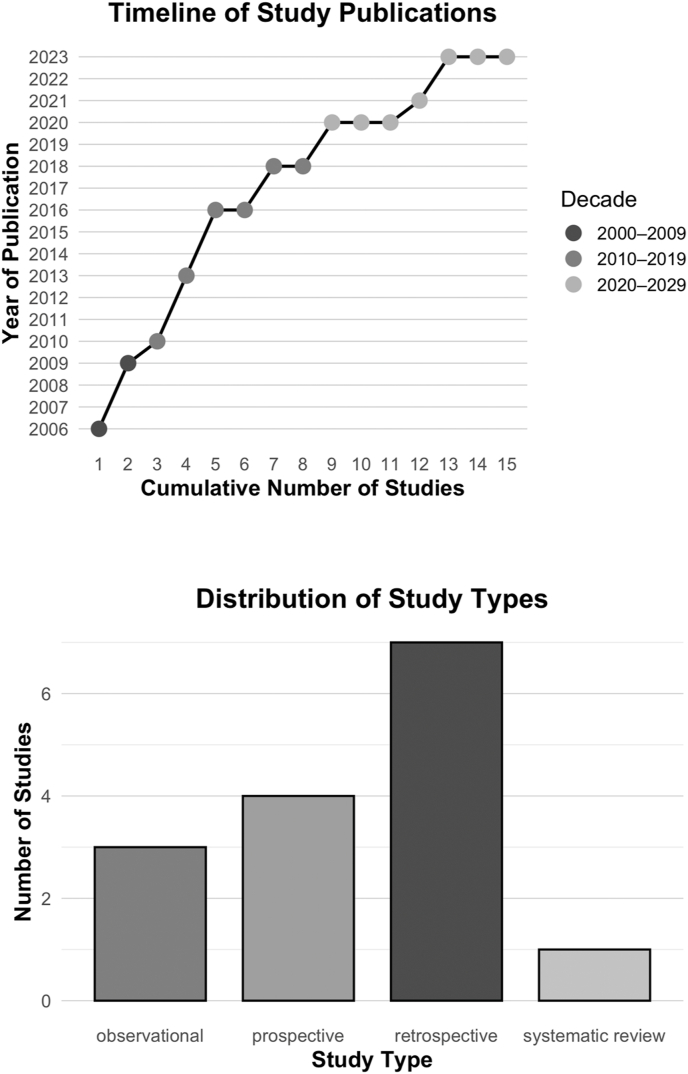
a) Number of articles per year of publication, b) study type (single-vs. multi-center) of the 15 selected studies published between 2006 and 2023.

15 selected articles were included in the final analysis. Most of these studies (n = 11) were published after 2015. Fig. 2 illustrates the trends in publications and study types (single-center vs. multi-center) per year from 2006 to 2023. 11 studies were single-center, whereas three studies were multi-center, and one study was a systematic review.

We further analyzed the types of studies included in this review, categorizing those as systematic reviews, retrospective, prospective, or observational studies. Most studies were conducted retrospectively (n = 7), 4 prospectively, while 3 were observational trials, and one systematic review.

**Fig. 3:**
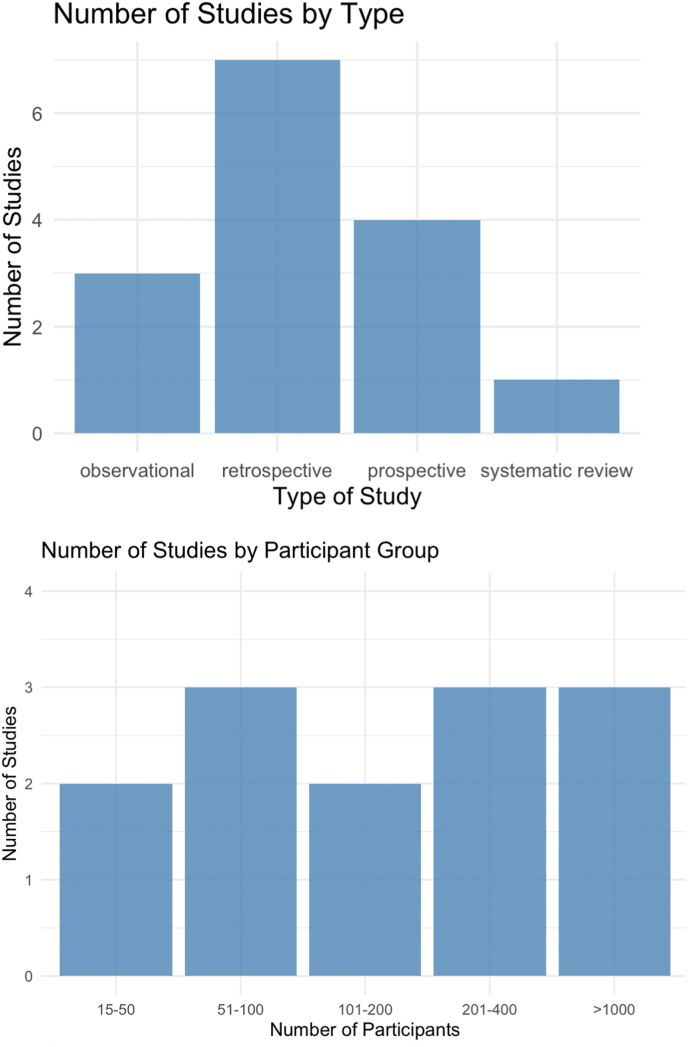
a) Study type (systematic review, retrospective, prospective or observational clinical study) and b) number of included patients (n = 15-50, 51-100, 101-200, 201-400, >1000) of the 14 selected clinical studies (systematic review excluded) published between 2006 and 2023.

We also analyzed the number of patients included in the 14 clinical studies (excluding the systematic review). The mean number of patients included was 228 (standard deviation: 5362), with a range from 15 to 18,529. We then grouped the number of patients into five categories (n = 15–50, 50–100, 100–200, 200–400, >1000). Fig. 3 presents the results on study type and the number of included patients.

The included studies focused on a range of surgical approaches. Eight studies examined spinal stenosis, spinal fusion or differences in sexual function following stenosis versus spinal fusion surgery (Fairbank, 2014; EuroQol, 1990; Malik et al., 2018; Holmberg et al., 2021; Hamilton et al., 2013). Two studies investigated lumbar disc herniation^,^ (Legato et al., 2016), and two focused on anterior lumbar interbody fusion (ALIF) surgeries (Grabala et al., 2023; Daniels et al., 2020). Additionally, one study focused on spinal deformity surgery (Gempt et al., 2010). Wuertz-Kozak et al. (2019) compares sexual sexual function in females after ALIF vs. total disc replacement, 93% of the patients were part of the ALIF group while only 7% in the total disc replacement group. Grabala et al. (2023) compares sexual dysfunction post posterior surgical correction. Here, there were two patient groups: severe scoliosis (45%) vs. moderate scoliosis (55%). In Cankaya et al. (2016) the patients were grouped into a short-segment posterior fixation group (50%) vs. long-segment fixation group (50%). On the other hand, Hägg et al. (Hagg et al., 2006) analyzed sexual function after ALIF in a non-surgical (68%) and surgical group (32%).

Twelve studies included both female and male patients. Two studies focused on sexual dysfunction in female patients only, and one focused solely on male patients. Most articles (n = 8) used question eight of the Oswestry Disability Index (ODI) questionnaire to assess sexual function. Other included questionnaires were the Female Sexual Function Index (FSFI)(Neijenhuijs et al., 2019) (n = 2), the Changes in Sexual Function Questionnaire-14 (CSFQ-14) (Clayton et al., 1997) (n = 1), the International Index of erectile function (IIEF-5) score of male patients (Warli et al., 2023) (n = 1) and self-tailored questionnaires (n = 2).

In the two studies researching anterior approaches, 1. Wuertz-Kozak et al. (2019) reported no changes in sexual function after ALIF or TDR, but an increase in sexual desire score after six months follow-up (Wuertz-Kozak et al., 2019). In Holmberg et al. (2021), 26.4% of patients reported a normal sex-life without pain preoperatively compared with 57.8% at 1 year. Preoperatively 10.5% reported that pain prevented any sex-life, compared with 5.3% at 1 year. In Sobański et al.(Sobanski et al., 2023), a statistically significantly higher level of sexual satisfaction was seen in the conservative-treatment group vs. surgical decompression. In Holmberg et al. (2020), at baseline, 84.5% of patients reported that sexual activity caused pain, and among these 68.8% reported an improvement at 1 year. On the contrary, in Hamilton et al. (2013) 24% of the patients had no sexual dysfunction postoperatively and 15%, 19%, and 42% had mild, moderate, and severe dysfunction, respectively. In Cankaya et al. (2016), SSPF was seen to cause a lesser decrease in the life quality score than LSPF. There was a decrease in the sexual function of male patients after thoracolumbar junction burst fractures. In Horst et al. (2016) at one year, 41% of the patients in the non-operative, 23% in the decompression group and 11% in the fusion group reported pain with sex life. In Kanayama et al. (2010) surgery improved sexual desire, frequency of sexual activity, and satisfaction in 85%, 88%, and 94%, respectively. In Hägg et al.(Hagg et al., 2006) the surgical cohort included four techniques: uninstrumented PLF (n = 68), instrumented PLF (n = 62), instrumented PLF + ALIF (n = 53), and instrumented PLF + PLIF (n = 18). For analysis, patients treated with PLF, PLF-I, and PLIF were grouped as “Posterior Fusion” (PF, n = 148), while those who underwent ALIF were classified as the “Anterior Fusion” (AF, n = 53) group. 53% of the patients reported an improvement of sexual function compared to 28% of the non-surgical patients. The improvement seemed to be independent of surgical approach. Finally, in Chan et al. (2020), at baseline, 81.7% had sex life impairment, compared to 73.0% at 2 years.

Two of the included studies focused exclusively on female patients. The first assessed sexual function following anterior lumbar surgery in women using the Female Sexual Function Index (FSFI). While no statistically significant change in the total FSFI score was observed over time, a significant increase in the FSFI desire score was noted between the preoperative value (2.95 ± 0.8) and the six-month follow-up (3.51 ± 0.6; P = 0.02) (Lim-Watson et al., 2022). The second study investigated changes in sexual function following a posterior approach for surgical correction for neglected idiopathic scoliosis using the Female Sexual Distress Scale (FSDS)(Derogatis et al., 2002). It reported a significant improvement in the mean preoperative FSDS score, from 13.6 to 6.8 over a follow-up of two years (Grabala et al., 2023).

The main characteristics of the 15 included studies are summarized in Table 1, including the primary author, year of publication, study type, number of participants, main research question, and key findings relevant to our study. The measurement systems to report on sexual dysfunction were depicted in Table 2. Also, a summarized result regarding sexual function for each of the 15 studies included in this review is shown in Table 3.

**Table 1:** Characteristics of the 15 studies examining sexual function after spinal surgery, including: year of publication, study type, number of participants, main question of the study and main relevant result. Abbreviations: ALIF: Anterior Lumbar interbody fusion; ASD: Adjacent Segment Disease; LSS: Lumbar Spinal Stenosis; NR: Sexual Life Not Relevant; PLIF: Posterior Lumbar Interbody Fusion; SLR: Sexual Life Relevant; SF: Sexual Function; TDR: Total Disc Replacement.

Paper	Year	Study Type	No. of Participants	Question	Measurement System
1.Wuertz-Kozak et al. (Wuertz-Kozak et al., 2019)	2018	Single center, retrospective	15 females (93% in ALIF group, 7% in TDR group)	SF after ALIF and lumbar TDR	FSFI (Female Sexual Function Index)
2.Holmberg et al. (Holmberg et al., 2021)	2021	Multi center, observational	9908	Pain during sex after LSS surgery	Oswestry Disability Index, EuroQol-5D
3.Grabala et al. (Grabala et al., 2023)	2023	Single center, retrospective	195 (45% in severe scoliosis and 55% in moderate scoliosis group)	SF after posterior surgical correction	Health-related quality of life (HRQoL)
4.Hamilton (Hamilton et al., 2013)	2013	Single center, retrospective	62	SF after spinal deformity surgery	Changes in Sexual Function Questionnaire-14 (CSFQ-14), Oswestry Disability Index (ODI), and 12-Item Short-Form Health Survey (SF-12)
5.Cankaya (Cankaya et al., 2016)	2016	Single center, prospective, randomized	24 (50% in SSPF group, 50% in LLPF group)	SF after short-segment posterior fixation (SSPF) vs. long-segment posterior fixation (LLPF)	EQ-5D and the International Index of erectile function (IIEF-5) score of male patients and the Female Sexual Function Index (FSFI)
6.Hägg et al. (Hagg et al., 2006)	2006	Single center, retrospective pilot study	284 (68% non-surgical, 32% surgical)	SF after ALIF	Oswestry Disability Index
7.Malic et al. (Malik et al., 2018)	2018	Systematic review	n.a.	SF after spinal surgery	Multiple measurement systems included
8.Daniels et al. (Daniels et al., 2020)	2020	Prospective, multicenter	368	SF after ASD surgery	
9.Berg et al. (Berg et al., 2009)	2009	Single center, randomized controlled trial	152	SF after FS (fusion surgery)	Oswestry Disability Index [ODI] 0-100), quality of life (EQ (5D) [EuroQol] 0-1), and answers on specific sexual function
10.Holmberg et al. (Holmberg et al., 2020)	2020	Multi center, observational	18,529	SF after LDH (lumbar disc herniation)	ODI, EuroQol-5D (EQ-5D)
11.Shimamura et al. (Shimamura et al., 2023)	2023	Single center, observational	35	SF after PLIF	Anonymous questionnaire concerning sexual desire, activity, and satisfaction before and after surgery
12. Horst et al. (Horst et al., 2016)	2016	Single center, restrospective	1235 (29% in NR group, 71% in SLR group)	SF after surgery for spinal stenosis (SPS) and degenerative spondylolisthesis (DS)	Oswestry Disability Index
13.Sobański et al. (Sobanski et al., 2023)	2023	Single center, prospective	96	SF after surgical decompression	Oswestry Low Back Pain Disability Questionnaire & Sexual Satisfaction Scale
14.Kanayama et al. (Kanayama et al., 2010)	2010	Single center, retrospective	90	SF after disc herniation surgery	Questionnaires concerning sexual desire, activity, adjustment, and satisfaction
15.Chan et al. (Chan et al., 2020)	2020	Single center, retrospective	218	SF after spondylolisthesis surgery	Oswestry Disability Index

**Table 2:** Example of the measurement systems for sexual dysfunction.

Measurement System	Number of Domains	Example
Oswestry Disability Index (ODI) questionnaire	10, one analyzing sexual function	No. 8: “My sex life is normal and causes no extra pain.” to “Pain prevents any sex life at all.”
Female Sexual Function Index (FSFI)	6	1. Desire 2. Arousal 3. Lubrication 4. Orgasm 5. Satisfaction 6. Pain
Female Sexual Distress Scale (FSDS)	12	Respondents rate how often they experienced sexually related personal distress in the past 30 days
Changes in Sexual Function Questionnaire-14 (CSFQ-14)	14	1. Sexual Desire/Frequency 2. Sexual Desire/Interest 3. Sexual Pleasure 4. Arousal/Excitement 5. Orgasm/Completion
International Index of erectile function (IIEF-5)	5	Questions to evaluate the severity of erectile dysfunction (ED)

**Table 3:** Summary of the results regarding sexual function of each of the 15 studies included in this review. Abbreviations: FSDS: Female Sexual Distress Scale; LSDI: Lumbar Stiffness Disability Index.

Paper	Results
1.Wuertz-Kozak et al. (Wuertz-Kozak et al., 2019)	No statistically significant change in the total Female Sexual Function Index (FSFI) in both groups.
2.Holmberg et al. (Holmberg et al., 2021)	Preoperatively 26.4% reported a normal sex-life without pain compared with 57.8% at 1 year. Preoperatively 10.5% reported that pain prevented any sex-life, compared with 5.3% at 1 year.
3.Grabala et al. (Grabala et al., 2023)	The preoperative FSDS score was 13.8 in the severe group and 31.4 in the moderate group. The FSDS score at FFU was 6.8 in the severe group and 4.5 in the moderate group.
4.Hamilton (Hamilton et al., 2013)	24% of the patients had no sexual dysfunction postoperatively and 15%, 19%, and 42% had mild, moderate, and severe dysfunction, respectively.
5.Cankaya (Cankaya et al., 2016)	SSPF was seen to cause a lesser decrease in the life quality score than LSPF. There was a decrease in the sexual function of male patients after thoracolumbar junction burst fractures.
6.Hägg et al. (Hagg et al., 2006)	53% of the patients reported an improvement of sexual function compared to 28% of the non-surgical patients.
7.Malic et al. (Malik et al., 2018)	There is a high incidence of initial pre-operative sexual dysfunction in patients before lumbar decompression, sexual function improved over time after.
8.Daniels et al. (Daniels et al., 2020)	LSDI sexual function scores averaged 1.7, which improved to 1.3 at 2-year follow-up.
9.Berg et al. (Berg et al., 2009)	After surgery, sex life improved in both groups, with a strong correlation to a reduction of low back pain.
10.Holmberg et al. (Holmberg et al., 2020)	At baseline, 84.5% of patients reported that sexual activity caused pain, and among these 68.8% reported an improvement at 1 year.
11.Shimamura et al. (Shimamura et al., 2023)	After surgery, Sexual desire, frequency of sexual activity and satisfaction did not regain after surgery in 94%, 93% and 92%, respectively.
12. Horst et al. (Horst et al., 2016)	At one year, 41%, 23% and 11% of the patients in the non-operative, decompression and fusion group reported pain with sex life, respectively.
13.Sobański et al. (Sobanski et al., 2023)	A statistically significantly higher level of sexual satisfaction was seen in the conservative-treatment group vs. surgical decompression.
14.Kanayama et al. (Kanayama et al., 2010)	Surgery improved sexual desire, frequency of sexual activity, and satisfaction in 85%, 88%, and 94%, respectively.
15.Chan et al. (Chan et al., 2020)	At baseline, 81.7% had sex life impairment, compared to 73.0% at 2 years.

## 4 Discussion

This study demonstrates that the evidence on the occurrence of sexual dysfunction following elective spine surgery remains scarce, although the number of published articles on this topic has been gradually increasing over recent years.

Most of the available research consists of low evidence, with only four prospective studies existing, two of which were randomized controlled trials. Additionally, the sample sizes in most studies were small. These findings highlight the need for future higher-quality research on this underexplored, yet clinically highly relevant topic.

Furthermore, the existing literature is heterogeneous, addressing sexual dysfunction in various contexts, including disc decompression, spinal fusion, anterior lumbar interbody fusion, and spinal deformity surgery. Consequently, the topic is approached from multiple perspectives. Overall, the studies indicate a general trend toward improved sexual function, reduced pain during sexual activity, and increased sexual satisfaction after surgery. For example, Holmberg et al. (2021) reported a 31.4% increase in the number of patients describing a normal sex life without pain one year postoperatively compared with preoperatively. In Kanayama et al. (2010), surgery improved sexual desire, frequency of sexual activity, and satisfaction in 85%, 88%, and 94% of patients, respectively. Grabala et al. (2023), Hägg et al.(Hagg et al., 2006), and Malic et al. (Malik et al., 2018) also support this trend, demonstrating postoperative improvements in sexual function over time.

However, this overall trend—suggesting no impairment of sexual function following spine surgery—should be interpreted cautiously within this broader context. Interestingly, two studies report a risk of sexual dysfunction after spinal surgery. In Hamilton et al. (2013), 24% of patients had no postoperative sexual dysfunction, whereas 15%, 19%, and 42% experienced mild, moderate, and severe dysfunction, respectively. Similarly, in Shimamura et al. (2023), sexual desire, frequency of sexual activity, and satisfaction did not return to preoperative levels in 94%, 93%, and 92% of patients, respectively.

While sexual physiology and the loss of anatomical function have been extensively investigated (Krassioukov and Elliott, 2017; Bhambhvani et al., 2022; He et al., 2006), only minimal research so far has focused on psychological and gender-specific changes in sexual function following spine surgery. In particular, studies on female sexual dysfunction after spine surgery across different pathologies and surgical techniques are scarce. For example, Goudman et al. reported that discussions on retrograde ejaculation and erectile dysfunction in males are more common than those on orgasmic alterations and lubrication disturbances in females (Goudman et al., 2024). Additionally, changes in spinal mobility and pelvic tilt following fusion procedures, along with stress and chronic pain, may contribute to a decline in sexual function among female patients (Hagg et al., 2006; Abdel et al., 2023).

Discussions on overall sexual health after spine surgery, including gender-specific differences, are often considered uncomfortable and therefore rarely conducted (Ricciardi et al., 2007; Barrett et al., 2022). For instance, Korse et al. revealed that 73% of neurosurgeons rarely or never discuss sexual health with their patients (Korse et al., 2016). Given the wide range of patient ages and socioeconomic backgrounds, variations in sexual health concerns and management approaches may arise (McCabe et al., 2016). It is also important to acknowledge the likely high number of unreported cases, as feelings of shame might prevent patients from speaking about such issues—both before and after surgery. Expanding our understanding of this multifaceted topic is essential for providing adequate patient education before surgery and comprehensive and refined care afterward (Salamanna et al., 2022; Kreuter et al., 2011).

Finally, there is controversy in the reported outcomes regarding sexual function after spine surgery, with some studies describing improvements and others reporting a decline in function. A critical limitation of the existing literature is that most studies do not differentiate between a loss of libido due to pain during sexual activity and physical limitations that directly impair sexual performance. Furthermore, many studies rely on general disability indices, such as the Oswestry Disability Index (ODI), to assess sexual dysfunction, despite the fact that the ODI was not specifically designed to evaluate the very nuanced aspects of sexual health.

With this study, we emphasize the need for a gender-specific, systematic assessment of sexual dysfunction following spine surgery to fully understand its impact on the patients' quality of life. A broader understanding of this topic will help healthcare providers offer tailored counseling and provide psychological, pharmaceutical, or surgical treatment options. Identifying patient groups at higher risk and proactively guiding them toward better sexual health would create opportunities for future research. We call for multicenter, longitudinal, prospective studies with gender-specific analyses, standardized pre- and postoperative assessment tools, patient-centered outcomes, and targeted treatment strategies to address and manage sexual dysfunction following spinal surgery.

## 5 Conclusion

This systematic review summarizes the current literature on the occurrence of sexual dysfunction following spine surgery and highlights that this important issue remains under-investigated. Most existing studies are heterogeneous, of low evidence quality, and rely primarily on general disability measures such as the Oswestry Disability Index (ODI), which was not specifically designed to evaluate the very nuanced aspects of sexual health. The overall trend suggesting no impairment of sexual function and even an improvement following spine surgery, should be interpreted cautiously with two studies reporting a risk of sexual dysfunction after spinal surgery. In the current body of literature, gender-specific differences and the multifaceted aspects of sexual health are insufficiently addressed, underscoring a significant gap in knowledge and comprehensive patient care. Future research should focus on multicenter, longitudinal studies that utilize validated, sexual function–specific instruments and include both pre- and postoperative assessments. Such an approach will improve the identification of at-risk patients and guide the development of tailored interventions.

## Declaration of competing interest

The authors declare that they have no known competing financial interests or personal relationships that could have appeared to influence the work reported in this paper.
